# Multiple Modes of Vitamin K Actions in Aging-Related Musculoskeletal Disorders

**DOI:** 10.3390/ijms20112844

**Published:** 2019-06-11

**Authors:** Kotaro Azuma, Satoshi Inoue

**Affiliations:** 1 Department of Systems Aging Science and Medicine, Tokyo Metropolitan Institute of Gerontology, 35-2 Sakae-cho, Itabashi-ku, Tokyo 173-0015, Japan; azumak@tmig.or.jp; 2 Division of Gene Regulation and Signal Transduction, Research Center for Genomic Medicine, Saitama Medical University, 1397-1 Yamane, Hidaka-shi, Saitama 350-1241, Japan

**Keywords:** γ-glutamyl carboxylase (GGCX), steroid and xenobiotic receptor (SXR), pregnane X receptor (PXR), osteoporosis, osteoarthritis, sarcopenia, osteocalcin, matrix Gla protein (MGP), Gla-rich protein (GRP)

## Abstract

Vitamin K is a fat-soluble vitamin that was originally found as an essential factor for blood coagulation. With the discovery of its role as a co-factor for γ-glutamyl carboxylase (GGCX), its function for blood coagulation was understood as the activation of several blood coagulation factors by their γ-carboxylation. Over the last two decades, other modes of vitamin K actions have been discovered, such as the regulation of transcription by activating the steroid and xenobiotic receptor (SXR), physical association to 17β-Hydroxysteroid dehydrogenase type 4 (17β-HSD4), covalent modification of Bcl-2 antagonist killer 1 (Bak), and the modulation of protein kinase A (PKA) activity. In addition, several epidemiological studies have revealed that vitamin K status is associated with some aging-related diseases including osteoporosis, osteoarthritis, and sarcopenia. Clinical studies on single nucleotide polymorphisms of GGCX suggested an association between higher GGCX activity and bone protective effect, while recent findings using conditional knockout mice implied that a contribution in protective effect for bone loss by GGCX in osteoblastic lineage was unclear. GGCX in other cell lineages or in other tissues might play a protective role for osteoporosis. Meanwhile, animal experiments by our groups among others revealed that SXR, a putative receptor for vitamin K, could be important in the bone metabolism. In terms of the cartilage protective effect of vitamin K, both GGCX- and SXR-dependent mechanisms have been suggested. In clinical studies on osteoarthritis, the γ-carboxylation of matrix Gla protein (MGP) and gla-rich protein (GRP) may have a protective role for the disease. It is also suggested that SXR signaling has protective role for cartilage by inducing family with sequence similarity 20a (*Fam20a*) expression in chondrocytes. In the case of sarcopenia, a high vitamin K status in plasma was associated with muscle strength, large muscle mass, and high physical performance in some observational studies. However, the basic studies explaining the effects of vitamin K on muscular tissue are limited. Further research on vitamin K will clarify new biological mechanisms which contribute to human longevity and health through the prevention and treatment of aging-related musculoskeletal disorders.

## 1. Introduction

**Figure 1:**
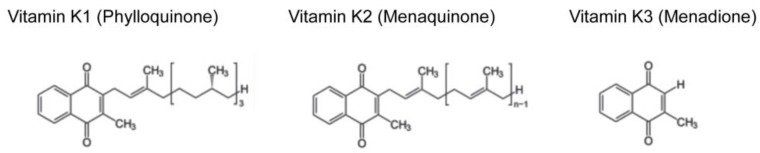
Molecular structures of the three forms of vitamin K. Vitamin K1, K2, and K3 share naphthoquinone ring, but differ in their side chains. Vitamin K1 has a phytyl side chain. Vitamin K2 has a side chain with varying number of isoprene units and called “MK-n” depending on the number of isoprene units. Vitamin K3 is a synthetic vitamin K without a side chain.

Vitamin K is a fat-soluble vitamin that is classified into three forms, namely vitamin K1 (phylloquinone), K2 (menaquinone), and K3 (menadione) (Figure 1). Among them, vitamin K1 and K2 are natural compounds, whereas vitamin K3 is an artificial one. Vitamin K1 is abundant in vegetables [1] and vitamin K2 is contained in fermented foods [2,3]. For example, Japanese fermented soybeans (called “natto”) contain high concentrations of vitamin K2 [2]. Vitamin K2 is referred as “MK-n” depending on the number of isoprene units in its side chain. For example, vitamin K2 abundant in “natto” is MK-7 [2], whereas MK-9, -10, and -11 are major forms of vitamin K2 in dairy food [3]. Although mammals do not have the ability to synthesize vitamin K2 de novo, they are able to convert vitamin K1 into MK-4 form of vitamin K2 by UbiA prenyltransferase domain containing protein l (UBIAD1) [4]. The *Ubiad1* knockout mice were embryonic lethal [5], suggesting essential physiological roles of vitamin K2, although the contribution of other substrates of UBIAD1 cannot be excluded. Vitamin K3 is widely used as a source of vitamin K in animal food. However, it is actually toxic in that it generates reactive oxygen species and has been banned from human consumption by the U.S. Food and Drug Administration since 1963.

Vitamin K was discovered by Danish biochemist Dr. Henrik Dam in 1929 as a fat-soluble diet substance necessary for blood coagulation [6]. The substance was named after the German word ‘Koagulationsvitamin’ by adopting the first letter of this word. Dr. Henrik Dam was awarded the Nobel Prize in Physiology or Medicine in 1943 with an American biochemist, Dr. Edward A, doisy, who determined the structure of both vitamin K1 and K2 [7]. It was not until the 1970s that part of the mechanism of vitamin K functions began to be clarified. Vitamin K was found to be a necessary factor for γ-carboxylation of some coagulation factors which is catalyzed by an enzyme called γ-glutamyl carboxylase (GGCX) [8,9].

Now, vitamin K is known to be involved in many biological processes other than blood coagulation. In addition, other modes of vitamin K action have been elucidated by studies from independent laboratories, including ours. In this review, we will introduce the mechanisms of vitamin K functions, including novel modes of action other than classical vitamin K action mediated by GGCX. Then, we discuss the functions of vitamin K in three aging-related musculoskeletal disorders; osteoporosis, osteoarthritis, and sarcopenia.

## 2. Vitamin K Function Mediated by γ-Carboxylation of Proteins

**Figure 2:**
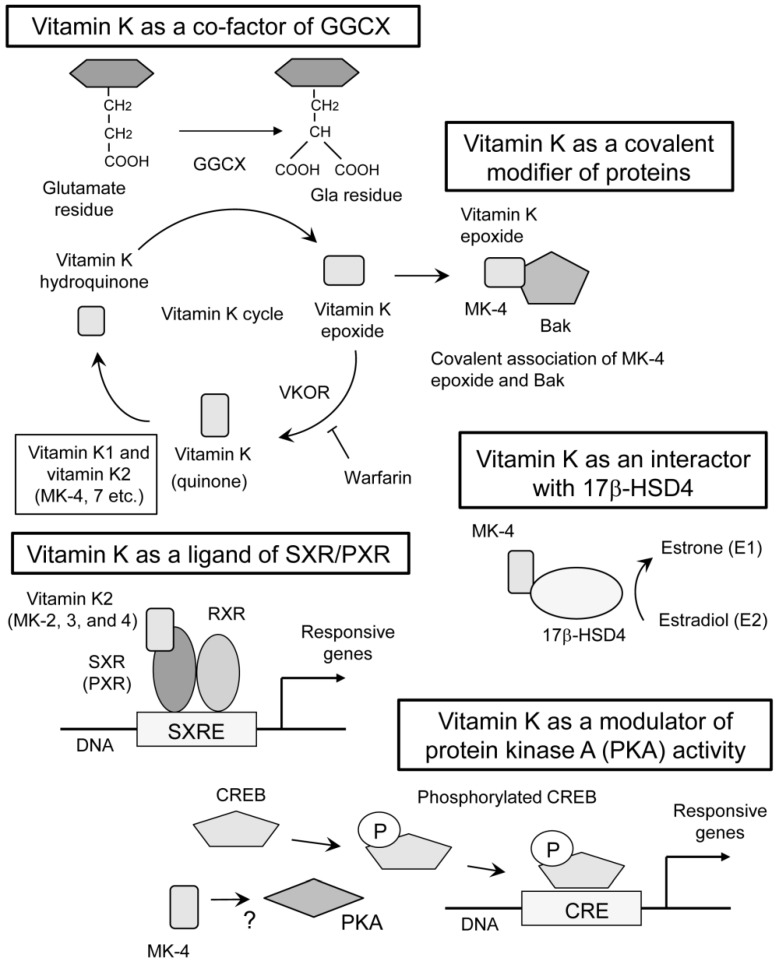
Multiple modes of vitamin K actions. The classical mechanism of vitamin K action is a co-factor for γ-glutamyl carboxylase (GGCX) [8,9,10,11]. This reaction requires cyclic use of vitamin K. Both vitamin K1 and K2 work in this mode of action. Vitamin K epoxide reductase (VKOR) is required for recycling vitamin K which is oxidized during γ-glutamyl carboxylation. Warfarin inhibits VKOR and vitamin K recycling, thereby suppressing GGCX activity. Vitamin K also functions as a ligand of steroid and xenobiotic receptor (SXR) and its murine homolog, pregnane X receptor (PXR) [14]. Some forms of vitamin K2 (MK-2, 3, and 4) are reported to have the ability to activate SXR [15], while vitamin K1 is not capable of activating SXR [16]. On vitamin K binding, SXR/PXR form heterodimers with 9-cis-retinoid acid receptor (RXR), and this complex binds to SXR-responsive elements (SXRE) within the regulatory regions of target genes. Covalent binding of vitamin K epoxide and a target protein, Bcl-2 antagonist killer 1 (Bak), is also proposed as a novel mode of vitamin K action [17]. This function is reported only in MK-4. Association of vitamin K with 17β-Hydroxysteroid dehydrogenase type 4 (17β-HSD4) can be different mode of vitamin K action [18], although mechanism of binding and regulation of enzymatic activity is unknown. Only MK-4 was used in this study. Finally, MK-4 activates protein kinase A (PKA) with unknown mechanism [19]. Typical substrate of PKA is CREB (cyclic AMP responsive element binding protein) and it binds to CRE (cyclic AMP responsive element) within the promoter or enhancer regions of target genes when CREB is phosphorylated. The arrows indicate conversion, association, or induction. The T bar indicates inhibition.

The earliest discovery of vitamin K function was its essential role in GGCX activity [8,9]. GGCX adds a carboxyl group to the gamma-position carbon of glutamate residues in the substrate proteins (Figure 2). The modified glutamate residue is called “Gla” residue. This reaction requires oxidization of vitamin K hydroquinone to vitamin K epoxide (Figure 2). This function of vitamin K was blocked with warfarin by inhibiting an enzyme called vitamin K epoxide reductase (VKOR) [10] which is necessary for cyclic use of vitamin K [11]. Coagulation factors II [8,9], VII [12], IX [13], and X [13] are well known substrates for GGCX. Activities of coagulation factors II, VII, IX and X are shown to be regulated by the γ-carboxylation of these glutamate residues, explaining the anti-coagulative function of warfarin [10].

**Figure 3:**
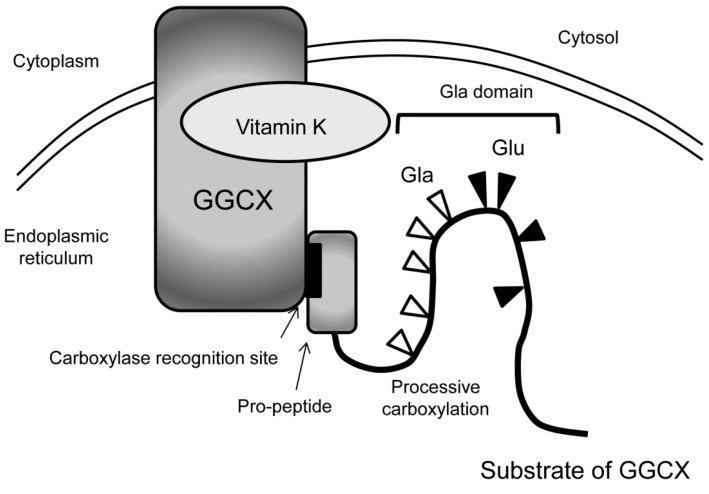
Processive γ-carboxylation of typical GGCX substrates. In the endoplasmic reticulum, typical substrates of GGCX bind with GGCX with the carboxylase recognition site in the pro-peptide at their N-termini (indicated by arrows). Then, multiple glutamate residues (Glu, black triangles) in the Gla domain are converted into Gla residues (white triangles) in processive manner.

GGCX resides in the membrane of the endoplasmic reticulum [20]. Typical substrates of GGCX have pro-peptides on their N-terminus and the substrates bind GGCX with carboxylase recognition site, a conserved motif within the pro-peptide [21]. Many of the substrates of GGCX have Gla domain next to the pro-peptide, where γ-carboxylation of multiple glutamate residues occurs in processive manner [22] (Figure 3). After the substrates are carboxylated, they become a mature protein by the dissection of pro-peptides.

As far as we know, 20 human proteins are found to be γ-carboxylated so far (Table 1). Among the γ-carboxylated proteins, we introduce 9 proteins which are shown to be related with musculoskeletal diseases, or potentially related to them.

Osteocalcin (also called bone Gla protein) [23,24] and matrix Gla protein (MGP) [31], which regulate the calcification of bone and cartilage, are substrates of GGCX. Their roles in osteoporosis or osteoarthritis will be disscussed later.

Growth arrest specific 6 (GAS6), which has multiple functions such as thrombus formation, inflammation, and cell proliferation, is also known as a substrate of GGCX [35]. GAS6 is shown to be a ligand for receptor tyrosine kinases such as Axl, Tyro3, and Mer. The γ-carboxylation of GAS6 is necessary for the activation of these receptors [36]. In relationship with the bone metabolism, GAS6 is shown to increase bone resorbing activity of osteoclast by binding Tyro3 expressed in osteoclast [37]. Another γ-carboxylated protein, protein S, was originally discovered as a protein involved in the fibrinolysis cascade [39]. Protein S, which is highly homologous to GAS6 [40], is also a ligand for Tyro3 [41], and activates osteoclasts [37]. In terms of cartilage metabolism, GAS6 is reported to have suppressive effect on chondrogenic differentiation [38].

In 2008, periostin and TGFβ induced (TGFBI) was reported to be members of γ-carboxylated proteins [42]. Periostin is involved in the maintenance of ligaments and bone development [43]. TGFBI is a protein homologous to periostin and has been shown to be involved in the maintenance of cornea and bone development [44,45]. In either periostin or TGFBI the significance of γ-carboxylation is not clarified. In fact, there is a study suggesting that the γ-carboxylation of periostin and TGFBI is doubtful [55].

In 2008, Gla-rich protein (GRP) which is also known as Upper zone of growth plate and cartilage matrix-associated protein (Ucma) was discovered from sturgeon cartilage and human fetal growth plate [46,47]. GRP is supposed to prevent the aberrant calcification of cartilage, which will be mentioned later.

Androgen receptor (AR) is a member of nuclear receptors. It mediates transcriptional regulation when the cells are stimulated with androgen. In 2017, it was shown that glutamate residue 2 of AR can be γ-carboxylated [49]. When the activity of VKOR was suppressed with warfarin or siRNAs, the transcriptional activity of AR was also suppressed. On the other hand, suppression of GGCX with siRNAs did not affect the transcriptional activity of AR [49], which makes it difficult to understand the biological significance of γ-carboxylation. In terms of bone metabolism, AR deficient mice display bone loss with enhanced bone resorption [50]. The suppressive effect of androgen on bone resorption was shown also in humans by a clinical study [51]. In the relationship between androgen and muscular tissue, the administration of testosterone is shown to increase skeletal muscle mass and strength in normal men [52]. In the animal model, AR deficient male mice displayed decreased skeletal muscle mass [53].

The final human protein carboxylated by GGCX is GGCX itself. In 1998, it was reported that three Gla residues in a GGCX molecule were carboxylated [54]. It was speculated that γ-carboxylation of GGCX may facilitate the release of GGCX substrates and suppressively regulate the processive γ-carboxylation of its substrates.

## 3. Vitamin K Function as a Ligand of SXR/PXR

In 2003, we discovered a completely different mechanism of vitamin K2 action mediated by transcriptional regulation. We showed that MK-4 form of vitamin K2 is one of the ligands for the nuclear receptor, steroid and xenobiotic receptor (SXR) and its murine ortholog, pregnane X receptor (PXR) [14,56] (Figure 2). This receptor is also known as NR1I2, according to standardized nomenclature designated by the nuclear receptor committee. Blumberg et al. cloned SXR/PXR as a novel nuclear receptor that is mainly expressed in the liver and intestine [57].

SXR/PXR is a ligand-dependent transcription factor and is activated by various drugs and xenobiotic compounds [58]. At the time of cloning, endogenous ligands of SXR/PXR had not been discovered. For that reason, SXR/PXR was originally classified as an orphan receptor. Now, secondary bile acids are known to be endogenous ligands for this receptor [59,60]. When SXR/PXR is activated by a ligand, it forms a heterodimer with 9-cis retinoid acid receptor (RXR). Then, this heterodimer is shown to bind with SXR-responsive elements (SXRE) in the regulatory regions of typical SXR/PXR-responsive genes [57] (Figure 2). Typical SXR/PXR-responsive genes include the drug metabolizing enzyme, *CYP3A4*, and the ABC family transporter, *MDR1*, which are involved in detoxification and drug excretion [61]. These responsive genes indicate that one of the roles of SXR/PXR function is a xenobiotic sensor. The expression of SXR/PXR has also been detected in the kidney, lung [62], and peripheral mononuclear cells [63].

Because of the difference of ligand-binding domain, the ligand specificity of SXR/PXR is known to be different among mammalian species. For example, rifampicin, an antibiotic drug, is an effective activator of human and rabbit SXR, while it has little activity on mouse SXR (PXR) or rat SXR. On the other hand, pregnane 16α-carbonitrile (PCN) activates mouse SXR (PXR) and rat SXR efficiently, while it does not activate human or rabbit SXR [15]. We reported that SXR is expressed in human osteoblastic cell lines and is activated by both rifampicin and MK-4 [14]. We also demonstrated that MK-4 can be a ligand for mouse SXR (PXR) by using osteoblastic cells derived from PXR-deficient mice [14]. Therefore, despite the difference of ligand specificity among mammalian species, vitamin MK-4 seems to activate both human SXR and murine SXR (PXR). Later, it was shown that MK-2 and MK-3 also activate SXR [64]. Meanwhile, it was shown that vitamin K1 itself is not capable of activating SXR [16], suggesting vitamin K1 potentially contribute to this mechanism of action after being converted into MK-4. The fact that some forms of vitamin K2 could be ligands for SXR has opened up the possibility that vitamin K is involved in many physiological and pathological processes through the regulation of PXR target genes.

## 4. Other Modes of Vitamin K Actions

In 2005, another interactor of vitamin K2 was discovered. Otsuka et al. showed that vitamin K2 binds 17β-Hydroxysteroid dehydrogenase type 4 (17β-HSD4) by mass spectrometric analysis with a pull-down assay using biotinylated MK-4 [18] (Figure 2). It is known that 17β-HSD4 is an enzyme converting estradiol (E2) to estrone (E1). They showed a vitamin K2-dependent decrease of DNA binding of estrogen receptor α, suggesting 17β-HSD4-mediated inactivation of E2 by converting it into E1. This study proposed another mode of vitamin K action with a physical interaction with proteins other than GGCX or SXR/PXR. However, the precise mechanism of regulation on the enzymatic activity is not clarified. Since estrogen signal is shown to protect bone tissues [65] and supposed to promote muscular endurance [66], activating 17β-HSD4 by MK-4 may not explain the apparent beneficial functions of vitamin K on musculoskeletal tissues stated below.

In 2007, we proposed the fourth mechanism of vitamin K action. In osteoblastic cells, MK-4 induced the gene expression of growth differentiation factor 15 (*GDF15*) and stanniocalcin 2 (*STC2*). The induction of these genes was considered to be mediated by neither GGCX nor SXR because the induction was not suppressed by treatment with rifampicin nor siRNA against SXR. Further investigation revealed that MK-4 induced the phosphorylation of protein kinase A (PKA) and that the inhibition of PKA suppressed the expression of *GDF15* and *STC2* in osteoblastic cells [19] (Figure 2). Although the precise mechanism of PKA activation is unknown, these results suggested the fourth mechanism of vitamin K action.

In 2013, a surprising action of vitamin K was reported. Karasawa et al. showed that pro-apoptotic protein Bcl-2 antagonist killer 1 (Bak) was covalently modified by epoxide form of MK-4 at cysteine 166 in Bak [17] (Figure 2). This modification of Bak functionally regulated apoptosis induction in human promyelocytic cell HL60 [17]. Bak regulates apoptosis also in osteoblasts. Attenuation of apoptosis by genetic deletion of Bak and Bax in osteoblasts increased femoral cancellous bone mass, whereas it also increased cortical porosity in aged mice [67]. This report suggests that the covalent modification of Bak with MK-4 may affect bone metabolism. In addition, if the covalent modification of proteins with vitamin K is a general phenomenon, other proteins affecting aging-related musculoskeletal disorders might be involved.

## 5. Roles of Vitamin K on Osteoporosis

The relationship of vitamin K and bone metabolism was suggested from a long time ago. In 1960, Bouckaert and Said reported that vitamin K enhanced the healing of fracture in rabbits [68]. The discovery of γ-carboxylated osteocalcin from bone tissues [23,24] also suggested a relationship. In 1985, Hart et al. reported that circulating levels of vitamin K1 in patients who had spinal or femoral neck fractures were significantly lower than those of age-matched control subjects [69]. This research was made possible by the invention of electrochemical detection for a low concentration of vitamin K in plasma by using high-performance liquid chromatography (HPLC). At the turn of the century, several large-scale epidemiological studies on the relationship between fracture risk and bone fracture were conducted. The epidemiological study conducted in North America revealed low vitamin K intake to be associated with an increased risk of hip fracture [70]. Interestingly, the association of vitamin K intake and bone mineral density (BMD) was not significantly observed in this study [70], suggesting its contribution to bone quality. The relationship of low vitamin K intake and hip fracture was also observed in Japanese observational study [71,72].

In Japan, MK-4 was approved for a drug for osteoporosis treatment in 1995 based on domestic clinical trials showing the efficacy on bone mineral density. Now, MK-4 is in use for osteoporosis treatment in several Asian countries. For the last two decades, interventional clinical trials were conducted throughout the world. Many trials used MK-4 as vitamin K treatment, MK-7 or vitamin K1 was also used in some trials as well. According to the most recent meta-analysis, a favorable effect of vitamin K on clinical fractures was statistically significant, although the effect on vertebral or hip fractures was not statistically significant [73]. Among the clinical trials, the largest one was conducted in Japan [74]. This study involved more than 4000 Japanese women with three years of intervention and a one-year follow-up period. This study failed to demonstrate the fracture-preventive effect of vitamin K2 (MK-4) in the whole group of subjects. However, the significant effect on new vertebral fractures was observed in the subgroup analysis of high-risk patients with at least five pre-existing vertebral fractures.

GGCX-dependent mechanism may exist in the bone protective role of vitamin K. We found a single nucleotide polymorphism (SNP) in GGCX significantly correlated with bone mineral density in elderly Japanese women [75]. This SNP involves amino acid substitution, where GGCX with higher enzymatic activity was coded by a beneficial allele with a higher bone mineral density. This result suggested that part of the bone protective effect of vitamin K could be mediated by GGCX activity. However, it is still a difficult process to clarify which substrates of GGCX are responsible for this effect. Osteocalcin, one of the substrates of GGCX [23,24], is specifically expressed in osteoblastic lineage [25,26] and regulates mineralization of bone [27]. The concentration of undercarboxylated form of osteocalcin (ucOC) in serum is positively correlated with fracture risk [28] and used as a biomarker for the indication of vitamin K treatment in Japan. In these circumstances, we first hypothesized that some GGCX substrate in osteoblasts, especially osteocalcin, may be responsible for the bone protective effect. To test this hypothesis, we generated osteoblast-specific GGCX-deficient mice [29]. As expected, serum ucOC was increased and carboxylated osteocalcin (cOC) was decreased compared with control mice. Notably, the bone mineral density was even increased in osteoblast-specific GGCX-deficient mice and mechanical strength was not impaired compared with control mice [29]. This result implied that GGCX in osteoblast may not contribute to the bone protective effect of vitamin K in this mouse model, and that the γ-carboxylation of osteocalcin might not be responsible for the effect. It is possible to speculate that GGCX activity in other cell lineages or in other tissues is responsible for the bone protective effect of vitamin K. In terms of the role of osteocalcin, it is noteworthy that osteocalcin-deficient mice were shown to have mechanically stronger bone than wild type mice [30], suggesting a decrease of cOC rather than increase of ucOC may have a bone strengthening effect.

The bone protective role of vitamin K may be explained by transcriptional regulation mediated by SXR/PXR. We found three SXR-dependent vitamin K-responsive genes, *Tsukushi*, *Matrillin2*, and *CD14* in the human osteoblastic cell line, MG63 cells stably overexpressing SXR [76]. *Tsukushi* and *Matrilin2* encode proteins related with extracellular matrix [77,78], and *CD14* encodes protein regulating bone turnover by inducing differentiation of B cells [79,80,81]. The bone phenotype of systemic *Pxr* knockout mice suggested the roles of SXR/PXR in the bone metabolism. We analyzed 4-month-old female *Pxr* knockout mice and found decreased bone mineral density, enhanced bone resorption, suppressed bone formation, and weaker mechanical strength in femoral bone from *Pxr* knockout mice compared with wild type mice [82]. Konno et al. analyzed *Pxr* knockout mice from different origin and reported lower bone mineral density in *Pxr* knockout mice [83]. They showed that *Slc34a2* is a PXR responsive gene in intestine and that serum concentration of inorganic phosphate was significantly decreased in *Pxr* knockout mice. Meanwhile, the serum levels of inorganic phosphate in *Pxr* knockout mice were not decreased in our observation [82], suggesting the mechanisms depend on the mouse strains and/or environment. In the case of humans, we found an SNP in the first intron of the *SXR* gene associated with the total body BMD of postmenopausal Japanese women [84]. This suggests that vitamin K actions mediated by SXR may have clinical significance.

## 6. Roles of Vitamin K on Osteoarthritis

Epidemiological studies in North America and Japan revealed the association of low vitamin K intake and the prevalence of osteoarthritis [85,86,87], suggesting the cartilage protective effect of vitamin K. This effect was further examined by an interventional trial with negative results [88], possibly due to short study period or weak effect of vitamin K on established osteoarthritis.

A mechanism of the protective effect of vitamin K on articular cartilage was proposed that the abnormal γ-carboxylation of proteins was involved in the aberrant calcification of cartilage. In a genome-wide association study, an SNP significantly associated with hand osteoarthritis was found near matrix Gla protein (*MGP*) gene [32], coding one of the substrates of GGCX. The risk allele was significantly associated with a lower expression of MGP in subchondral bone. It is notable that *Mgp* knockout mice displayed inappropriate calcification of cartilages [33], and that human chondrocytes derived from patient of osteoarthritis produced less carboxylated MGP (cMGP) and more undercarboxylated MGP (ucMGP) [34]. Taken together it is likely that cMGP has a beneficial effect on preventing osteoarthritis by inhibiting the calcification of articular cartilage. Another candidate molecule which can explain protective effect of vitamin K on cartilage is Gla-rich protein (GRP). In osteoarthritic cartilage undercarboxylated GRP (ucGRP) was more evident than carboxylated GRP (cGRP), and both ucGRP and cGRP co-localized with ectopic calcification [48]. This observation suggested γ-carboxylation of GRP is important for inhibiting calcification of cartilage tissue.

The cartilage protective effect of vitamin K may also be explained by transcriptional regulation mediated by SXR/PXR. We found that *Pxr* knockout mice displayed aging-dependent wearing of articular cartilage of knee joints, resembling development of osteoarthritic lesion [89]. We identified an SXR-dependent vitamin K (MK-4)-responsive gene, *Fam20a* (family with sequence similarity 20a) using ATDC5 chondrocytic cells overexpressing human SXR [89]. Although the role of FAM20A in cartilage tissue is unknown, we speculate that FAM20A may regulate phosphorylation of certain extracellular matrix protein like other members of Fam20 family proteins [90,91].

## 7. Roles of Vitamin K on Sarcopenia

In 1988, the word ‘sarcopenia’ was invented by combining Greek words meaning ‘muscle’ and ‘loss’ [92]. However, it was not until 2010 when the operational definition of sarcopenia began to be proposed to familiarise people with pathological muscle loss [93]. Therefore, sarcopenia is an emerging clinical entity which requires attention to prevent locomotor disabilities. In 2016, sarcopenia was added to the list of International Classification of Diseases (ICD)-10, indicating that sarcopenia is now internationally regarded as a disease. In this decade, several definitions and diagnostic criteria have been proposed throughout the world, and clinical or basic studies on sarcopenia have been undertaken. Currently, more emphasis is put on muscle strength and physical performance rather than muscle mass [94], because muscle strength and physical performance are directly associated with disabilities of patients.

Some clinical observational studies suggest that vitamin K status is related to physical performance. One large clinical study conducted in the United States analyzed 1089 community-dwelling older adults, and reported that a higher plasma concentration of vitamin K1 was associated with higher SPPB (short physical performance battery; including the evaluation of gait speed, chair stand, and balance test) scores in both cross-sectional and longitudinal analyses [95]. More than half (67%) of the participants were female in this study, although the subgroup analysis of each gender was not performed. Another large observational study, in which 633 community-dwelling adults aged 55–56 years old participated, revealed that a higher vitamin K status was associated with a higher physical performance score (assessed with walk test, chair stand test, and cardigan putting on and taking off time) only in women [96]. Stronger handgrip power and larger calf circumference were associated with higher vitamin K status in both genders. According to a randomized controlled study originally aimed to evaluate the effect of vitamin K1 supplementation on bone loss and coronary artery calcification, three-year vitamin K1 supplementation did not change appendicular lean mass, which reflects muscle mass of arms and legs [97]. Unfortunately, physical performance was not assessed in this study. Another control study with six-month vitamin K2 (MK-7) supplementation did evaluate physical performance but showed no significant effect of vitamin K on handgrip strength and SPPB score [98].

Considering the results of several clinical studies introduced above, vitamin K may have a beneficial effect on muscle quality reflected by physical performance scores rather than muscle mass. As far as we know, there is no convincing basic study explaining the mechanisms of vitamin K functions in the skeletal muscle tissue. Further clinical and basic studies should be required for a detailed discussion on these matters.

## 8. Conclusions

In the present review, we described multiple mechanisms of vitamin K functions and their involvement in aging-related musculoskeletal disorders as examples for the biological significance of vitamin K beyond its roles in blood coagulation. In a super-aged society, musculoskeletal disorders are the leading cause of disabilities requiring health care. Therefore, osteoporosis, osteoarthritis and sarcopenia are becoming more important issues as obstacles to expanding a healthy life span in an aging society. We sincerely hope that studies on vitamin K lead to the discovery of new biological mechanisms and targets for disease prevention and treatment, and eventually contribute to human longevity and health by the clinical application of these discoveries.

## Figures and Tables

**Table 1:** Musculoskeletal disease-related γ-carboxylated proteins. Among 20 kinds of proteins known to be γ-carboxylated, 9 proteins are related or possibly related to musculoskeletal diseases.

Proteins	Expression	Function	References
Osteocalcin (Bone Gla protein, BGP)	Osteoblast	Regulation of bone calcification, Regulation of glucose metabolism? Enhancing male fertility?	[23,24,25,26,27,28,29,30]
Matrix Gla protein (MGP)	Chondrocyte, vascular smooth muscle cell, etc.	Regulation of calcification	[31,32,33,34]
Growth arrest specific-6 (GAS6)	Lung, heart, kidney, intestine, endothelium, vascular smooth muscle cell, bone marrow, osteoblast, osteoclast, monocyte, etc.	Thrombus formation; Inflammation; Cell proliferation; Enhancing osteoclast activity	[35,36,37,38]
Protein S	Liver, endothelium, monocyte, etc.	Anti-coagulation; Enhancing osteoclast activity	[37,39,40,41]
Periostin	Periosteum, periodontal ligament, heart valve, adrenal gland, lung, thyroid, intestine, ovary, testis, prostate	Maintenance of periodontal ligament; Bone development and maintenance	[42,43]
TGFβ induced (TGFBI)	Bone, joint, skin, cornea, kidney.	Bone development; Maintenance of cornea	[42,44,45]
Gla-rich protein (GRP)/Upper zone of growth plate and cartilage matrix associated protein (Ucma)	Chondrocyte, osteoblast, osteocyte, vascular smooth muscle cell, skin	Suppression of inflammation; Suppression of calcification in blood vessels and articular cartilages	[46,47,48]
Androgen receptor	Testis, prostate, muscle, brain, prostate cancer	Induction of male sexual characteristics; Promotion of prostate cancer; Bone protection; Enhancing skeletal muscle mass	[49,50,51,52,53]
γ-glutamyl carboxylase (GGCX)	Systemic (High expression in liver)	γ-carboxylation of proteins	[54]

## References

[1] Suttie J.W., Booth S.L. (2011). Vitamin K. Adv Nutr..

[2] Tarvainen M., Fabritius M., Yang B. (2019). Determination of vitamin K composition of fermented food. Food Chem..

[3] Fu X., Harshman S.G., Shen X., Haytowitz D.B., Karl J.P., Wolfe B.E., Booth S.L. (2017). Multiple Vitamin K Forms Exist in Dairy Foods. Curr. Dev. Nutr..

[4] Nakagawa K., Hirota Y., Sawada N., Yuge N., Watanabe M., Uchino Y., Okuda N., Shimomura Y., Suhara Y., Okano T. (2010). Identification of UBIAD1 as a novel human menaquinone-4 biosynthetic enzyme. Nature.

[5] Nakagawa K., Sawada N., Hirota Y., Uchino Y., Suhara Y., Hasegawa T., Amizuka N., Okamoto T., Tsugawa N., Kamao M. (2014). Vitamin K2 biosynthetic enzyme, UBIAD1 is essential for embryonic development of mice. PLoS ONE.

[6] Dam H. (1935). The antihemorrhagic vitamin of the chick: Occurrence and chemical nature. Nature.

[7] Doisy E.A., Binkley S.B., Thayer S.A., McKee R.W. (1940). VITAMIN K. Science.

[8] Nelsestuen G.L., Zytkovicz T.H., Howard J.B. (1974). The mode of action of vitamin K. Identification of gamma-carboxyglutamic acid as a component of prothrombin. J. Biol. Chem..

[9] Stenflo J., Fernlund P., Egan W., Roepstorff P. (1974). Vitamin K dependent modifications of glutamic acid residues in prothrombin. Proc. Natl. Acad. Sci. USA.

[10] Czogalla K.J., Biswas A., Höning K., Hornung V., Liphardt K., Watzka M., Oldenburg J. (2017). Warfarin and vitamin K compete for binding to Phe55 in human VKOR. Nat. Struct. Mol. Biol..

[11] Stafford D.W. (2005). The vitamin K cycle. J. Thromb Haemost..

[12] Dissing C., Persson E. (2017). Factor VII Tokushima (Cys22→Gly) is not -carboxylated due to a disrupted γ-carboxylase recognition site. Thromb Res..

[13] Bucher D., Nebelin E., Thomsen J., Stenflo J. (1976). Identification of gamma-carboxyglutamic acid residues in bovine factors IX and X, and in a new vitamin K-dependent protein. FEBS Lett..

[14] Tabb M.M., Sun A., Zhou C., Grün F., Errandi J., Romero K., Pham H., Inoue S., Mallick S., Lin M. (2003). Vitamin K2 regulation of bone homeostasis is mediated by the steroid and xenobiotic receptor SXR. J. Biol. Chem..

[15] Jones S.A., Moore L.B., Shenk J.L., Wisely G.B., Hamilton G.A., McKee D.D., Tomkinson N.C., LeCluyse E.L., Lambert M.H., Willson T.M. (2000). The pregnane X receptor: A promiscuous xenobiotic receptor that has diverged during evolution. Mol. Endocrinol..

[16] Suhara Y., Hanada N., Okitsu T., Sakai M., Watanabe M., Nakagawa K., Wada A., Takeda K., Takahashi K., Tokiwa H. (2012). Structure-activity relationship of novel menaquinone-4 analogues: Modification of the side chain affects their biological activities. J. Med. Chem..

[17] Karasawa S., Azuma M., Kasama T., Sakamoto S., Kabe Y., Imai T., Yamaguchi Y., Miyazawa K., Handa H. (2013). Vitamin K2 covalently binds to Bak and induces Bak-mediated apoptosis. Mol. Pharmacol..

[18] Otsuka M., Kato N., Ichimura T., Abe S., Tanaka Y., Taniguchi H., Hoshida Y., Moriyama M., Wang Y., Shao R.X. (2005). Vitamin K2 binds 17beta-hydroxysteroid dehydrogenase 4 and modulates estrogen metabolism. Life Sci..

[19] Ichikawa T., Horie-Inoue K., Ikeda K., Blumberg B., Inoue S. (2007). Vitamin K2 induces phosphorylation of protein kinase A and expression of novel target genes in osteoblastic cells. J. Mol. Endocrinol..

[20] Tie J., Wu S.M., Jin D., Nicchitta C.V., Stafford D.W. (2000). A topological study of the human gamma-glutamyl carboxylase. Blood.

[21] Kulman J.D., Harris J.E., Xie L., Davie E.W. (2001). Identification of two novel transmembrane gamma-carboxyglutamic acid proteins expressed broadly in fetal and adult tissues. Proc. Natl. Acad. Sci. USA.

[22] Morris D.P., Stevens R.D., Wright D.J., Stafford D.W. (1995). Processive post-translational modification. Vitamin K-dependent carboxylation of a peptide substrate. J. Biol. Chem..

[23] Hauschka P.V., Lian J.B., Gallop P.M. (1975). Direct identification of the calcium-binding amino acid, gamma-carboxyglutamate, in mineralized tissue. Proc. Natl. Acad. Sci. USA.

[24] Price P.A., Otsuka A.A., Poser J.W., Kristaponis J., Raman N. (1976). Characterization of a gamma-carboxyglutamic acid-containing protein from bone. Proc. Natl. Acad. Sci. USA.

[25] Weinreb M., Shinar D., Rodan G.A. (1990). Different pattern of alkaline phosphatase, osteopontin, and osteocalcin expression in developing rat bone visualized by in situ hybridization. J. Bone Miner. Res..

[26] Zhang M., Xuan S., Bouxsein M.L., von Stechow D., Akeno N., Faugere M.C., Malluche H., Zhao G., Rosen C.J., Efstratiadis A. (2002). Osteoblast-specific knockout of the insulin-like growth factor (IGF) receptor gene reveals an essential role of IGF signaling in bone matrix mineralization. J. Biol. Chem..

[27] Hoang Q.Q., Sicheri F., Howard A.J., Yang D.S. (2003). Bone recognition mechanism of porcine osteocalcin from crystal structure. Nature.

[28] Szulc P., Chapuy M.C., Meunier P.J., Delmas P.D. (1993). Serum undercarboxylated osteocalcin is a marker of the risk of hip fracture in elderly women. J. Clin. Investig..

[29] Azuma K., Shiba S., Hasegawa T., Ikeda K., Urano T., Horie-Inoue K., Ouchi Y., Amizuka N., Inoue S. (2015). Osteoblast-Specific γ-Glutamyl Carboxylase-Deficient Mice Display Enhanced Bone Formation With Aberrant Mineralization. J. Bone Miner. Res..

[30] Ducy P., Desbois C., Boyce B., Pinero G., Story B., Dunstan C., Smith E., Bonadio J., Goldstein S., Gundberg C. (1996). Increased bone formation in osteocalcin-deficient mice. Nature.

[31] Price P.A., Urist M.R., Otawara Y. (1983). Matrix Gla protein, a new gamma-carboxyglutamic acid-containing protein which is associated with the organic matrix of bone. Biochem. Biophys. Res. Commun..

[32] den Hollander W., Boer C.G., Hart D.J., Yau M.S., Ramos Y.F.M., Metrustry S., Broer L., Deelen J., Cupples L.A., Rivadeneira F. (2017). Genome-wide association and functional studies identify a role for matrix Gla protein in osteoarthritis of the hand. Ann. Rheum. Dis..

[33] Luo G., Ducy P., McKee M.D., Pinero G.J., Loyer E., Behringer R.R., Karsenty G. (1997). Spontaneous calcification of arteries and cartilage in mice lacking matrix GLA protein. Nature.

[34] Wallin R., Schurgers L.J., Loeser R.F. (2010). Biosynthesis of the vitamin K-dependent matrix Gla protein (MGP) in chondrocytes: A fetuin-MGP protein complex is assembled in vesicles shed from normal but not from osteoarthritic chondrocytes. Osteoarthr. Cartil..

[35] Varnum B.C., Young C., Elliott G., Garcia A., Bartley T.D., Fridell Y.W., Hunt R.W., Trail G., Clogston C., Toso R.J. (1995). Axl receptor tyrosine kinase stimulated by the vitamin K-dependent protein encoded by growth-arrest-specific gene 6. Nature.

[36] Nagata K., Ohashi K., Nakano T., Arita H., Zong C., Hanafusa H., Mizuno K. (1996). Identification of the product of growth arrest-specific gene 6 as a common ligand for Axl, Sky, and Mer receptor tyrosine kinases. J. Biol. Chem..

[37] Nakamura Y.S., Hakeda Y., Takakura N., Kameda T., Hamaguchi I., Miyamoto T., Kakudo S., Nakano T., Kumegawa M., Suda T. (1998). Tyro 3 receptor tyrosine kinase and its ligand, Gas6, stimulate the function of osteoclasts. Stem Cells.

[38] Motomura H., Niimi H., Sugimori K., Ohtsuka T., Kimura T., Kitajima I. (2007). Gas6, a new regulator of chondrogenic differentiation from mesenchymal cells. Biochem. Biophys. Res. Commun..

[39] Stenflo J., Jönsson M. (1979). Protein S, a new vitamin K-dependent protein from bovine plasma. FEBS Lett..

[40] Manfioletti G., Brancolini C., Avanzi G., Schneider C. (1993). The protein encoded by a growth arrest-specific gene (gas6) is a new member of the vitamin K-dependent proteins related to protein S, a negative coregulator in the blood coagulation cascade. Mol. Cell Biol..

[41] Stitt T.N., Conn G., Gore M., Lai C., Bruno J., Radziejewski C., Mattsson K., Fisher J., Gies D.R., Jones P.F. (1995). The anticoagulation factor protein S and its relative, Gas6, are ligands for the Tyro 3/Axl family of receptor tyrosine kinases. Cell.

[42] Coutu D.L., Wu J.H., Monette A., Rivard G.E., Blostein M.D., Galipeau J. (2008). Periostin, a member of a novel family of vitamin K-dependent proteins, is expressed by mesenchymal stromal cells. J. Biol. Chem..

[43] Rios H., Koushik S.V., Wang H., Wang J., Zhou H.M., Lindsley A., Rogers R., Chen Z., Maeda M., Kruzynska-Frejtag A. (2005). Periostin null mice exhibit dwarfism, incisor enamel defects, and an early-onset periodontal disease-like phenotype. Mol. Cell Biol..

[44] Yu H., Wergedal J.E., Zhao Y., Mohan S. (2012). Targeted disruption of TGFBI in mice reveals its role in regulating bone mass and bone size through periosteal bone formation. Calcif Tissue Int..

[45] Stewart H., Black G.C., Donnai D., Bonshek R.E., McCarthy J., Morgan S., Dixon M.J., Ridgway A.A. (1999). A mutation within exon 14 of the TGFBI (BIGH3) gene on chromosome 5q31 causes an asymmetric, late-onset form of lattice corneal dystrophy. Ophthalmology.

[46] Tagariello A., Luther J., Streiter M., Didt-Koziel L., Wuelling M., Surmann-Schmitt C., Stock M., Adam N., Vortkamp A., Winterpacht A. (2008). Ucma--A novel secreted factor represents a highly specific marker for distal chondrocytes. Matrix Biol..

[47] Viegas C.S., Simes D.C., Laizé V., Williamson M.K., Price P.A., Cancela M.L. (2008). Gla-rich protein (GRP), a new vitamin K-dependent protein identified from sturgeon cartilage and highly conserved in vertebrates. J. Biol. Chem..

[48] Rafael M.S., Cavaco S., Viegas C.S., Santos S., Ramos A., Willems B.A., Herfs M., Theuwissen E., Vermeer C., Simes D.C. (2014). Insights into the association of Gla-rich protein and osteoarthritis, novel splice variants and γ-carboxylation status. Mol. Nutr. Food Res..

[49] Tew B.Y., Hong T.B., Otto-Duessel M., Elix C., Castro E., He M., Wu X., Pal S.K., Kalkum M., Jones J.O. (2017). Vitamin K epoxide reductase regulation of androgen receptor activity. Oncotarget.

[50] Kawano H., Sato T., Yamada T., Matsumoto T., Sekine K., Watanabe T., Nakamura T., Fukuda T., Yoshimura K., Yoshizawa T. (2003). Suppressive function of androgen receptor in bone resorption. Proc. Natl. Acad. Sci. USA.

[51] Falahati-Nini A., Riggs B.L., Atkinson E.J., O’Fallon W.M., Eastell R., Khosla S. (2000). Relative contributions of testosterone and estrogen in regulating bone resorption and formation in normal elderly men. J. Clin. Investig..

[52] Bhasin S., Storer T.W., Berman N., Callegari C., Clevenger B., Phillips J., Bunnell T.J., Tricker R., Shirazi A., Casaburi R. (1996). The effects of supraphysiologic doses of testosterone on muscle size and strength in normal men. N. Engl. J. Med..

[53] MacLean H.E., Chiu W.S., Notini A.J., Axell A.M., Davey R.A., McManus J.F., Ma C., Plant D.R., Lynch G.S., Zajac J.D. (2008). Impaired skeletal muscle development and function in male, but not female, genomic androgen receptor knockout mice. FASEB J..

[54] Berkner K.L., Pudota B.N. (1998). Vitamin K-dependent carboxylation of the carboxylase. Proc. Natl. Acad. Sci. USA.

[55] Annis D.S., Ma H., Balas D.M., Kumfer K.T., Sandbo N., Potts G.K., Coon J.J., Mosher D.F. (2015). Absence of Vitamin K-Dependent γ-Carboxylation in Human Periostin Extracted from Fibrotic Lung or Secreted from a Cell Line Engineered to Optimize γ-Carboxylation. PLoS ONE.

[56] Sultana H., Watanabe K., Rana M.M., Takashima R., Ohashi A., Komai M., Shirakawa H. (2018). Effects of Vitamin K_2_ on the Expression of Genes Involved in Bile Acid Synthesis and Glucose Homeostasis in Mice with Humanized PXR. Nutrients.

[57] Blumberg B., Sabbagh W., Juguilon H., Bolado J., van Meter C.M., Ong E.S., Evans R.M. (1998). SXR, a novel steroid and xenobiotic-sensing nuclear receptor. Genes Dev..

[58] Zhou C., Verma S., Blumberg B. (2009). The steroid and xenobiotic receptor (SXR), beyond xenobiotic metabolism. Nucl. Recept. Signal..

[59] Staudinger J.L., Goodwin B., Jones S.A., Hawkins-Brown D., MacKenzie K.I., LaTour A., Liu Y., Klaassen C.D., Brown K.K., Reinhard J. (2001). The nuclear receptor PXR is a lithocholic acid sensor that protects against liver toxicity. Proc. Natl. Acad. Sci. USA.

[60] Xie W., Radominska-Pandya A., Shi Y., Simon C.M., Nelson M.C., Ong E.S., Waxman D.J., Evans R.M. (2001). An essential role for nuclear receptors SXR/PXR in detoxification of cholestatic bile acids. Proc. Natl. Acad. Sci. USA.

[61] Synold T.W., Dussault I., Forman B.M. (2001). The orphan nuclear receptor SXR coordinately regulates drug metabolism and efflux. Nat. Med..

[62] Miki Y., Suzuki T., Tazawa C., Blumberg B., Sasano H. (2005). Steroid and xenobiotic receptor (SXR), cytochrome P450 3A4 and multidrug resistance gene 1 in human adult and fetal tissues. Mol. Cell Endocrinol..

[63] Albermann N., Schmitz-Winnenthal F.H., Z’graggen K., Volk C., Hoffmann M.M., Haefeli W.E., Weiss J. (2005). Expression of the drug transporters MDR1/ABCB1, MRP1/ABCC1, MRP2/ABCC2, BCRP/ABCG2, and PXR in peripheral blood mononuclear cells and their relationship with the expression in intestine and liver. Biochem. Pharmacol..

[64] Suhara Y., Watanabe M., Motoyoshi S., Nakagawa K., Wada A., Takeda K., Takahashi K., Tokiwa H., Okano T. (2011). Synthesis of new vitamin K analogues as steroid and xenobiotic receptor (SXR) agonists: Insights into the biological role of the side chain part of vitamin K. J. Med. Chem..

[65] Anderson G.L., Limacher M., Assaf A.R., Bassford T., Beresford S.A., Black H., Bonds D., Brunner R., Brzyski R., Caan B. (2004). Effects of conjugated equine estrogen in postmenopausal women with hysterectomy: The Women’s Health Initiative randomized controlled trial. JAMA.

[66] Nagai S., Ikeda K., Horie-Inoue K., Shiba S., Nagasawa S., Takeda S., Inoue S. (2016). Estrogen modulates exercise endurance along with mitochondrial uncoupling protein 3 downregulation in skeletal muscle of female mice. Biochem. Biophys. Res. Commun..

[67] Jilka R.L., O’Brien C.A., Roberson P.K., Bonewald L.F., Weinstein R.S., Manolagas S.C. (2014). Dysapoptosis of osteoblasts and osteocytes increases cancellous bone formation but exaggerates cortical porosity with age. J. Bone Miner. Res..

[68] Bouckaert J.H., Said A.H. (1960). Fracture healing by vitamin K. Nature.

[69] Hart J.P., Shearer M.J., Klenerman L., Catterall A., Reeve J., Sambrook P.N., Dodds R.A., Bitensky L., Chayen J. (1985). Electrochemical detection of depressed circulating levels of vitamin K1 in osteoporosis. J. Clin. Endocrinol. Metab..

[70] Booth S.L., Tucker K.L., Chen H., Hannan M.T., Gagnon D.R., Cupples L.A., Wilson P.W., Ordovas J., Schaefer E.J., Dawson-Hughes B. (2000). Dietary vitamin K intakes are associated with hip fracture but not with bone mineral density in elderly men and women. Am. J. Clin. Nutr..

[71] Kaneki M., Hodges S.J., Hosoi T., Fujiwara S., Lyons A., Crean S.J., Ishida N., Nakagawa M., Takechi M., Sano Y. (2001). Japanese fermented soybean food as the major determinant of the large geographic difference in circulating levels of vitamin K2: Possible implications for hip-fracture risk. Nutrition.

[72] Yaegashi Y., Onoda T., Tanno K., Kuribayashi T., Sakata K., Orimo H. (2008). Association of hip fracture incidence and intake of calcium, magnesium, vitamin D, and vitamin K. Eur. J. Epidemiol..

[73] Mott A., Bradley T., Wright K., Cockayne E.S., Shearer M.J., Adamson J., Lanham-New S.A., Torgerson D.J. (2019). Effect of vitamin K on bone mineral density and fractures in adults: An updated systematic review and meta-analysis of randomised controlled trials. Osteoporos Int..

[74] Inoue T., Fujita T., Kishimoto H., Makino T., Nakamura T., Nakamura T., Sato T., Yamazaki K. (2009). Randomized controlled study on the prevention of osteoporotic fractures (OF study): A phase IV clinical study of 15-mg menatetrenone capsules. J. Bone Miner. Metab..

[75] Kinoshita H., Nakagawa K., Narusawa K., Goseki-Sone M., Fukushi-Irie M., Mizoi L., Yoshida H., Okano T., Nakamura T., Suzuki T. (2007). A functional single nucleotide polymorphism in the vitamin-K-dependent gamma-glutamyl carboxylase gene (Arg325Gln) is associated with bone mineral density in elderly Japanese women. Bone.

[76] Ichikawa T., Horie-Inoue K., Ikeda K., Blumberg B., Inoue S. (2006). Steroid and xenobiotic receptor SXR mediates vitamin K2-activated transcription of extracellular matrix-related genes and collagen accumulation in osteoblastic cells. J. Biol. Chem..

[77] Ohta K., Lupo G., Kuriyama S., Keynes R., Holt C.E., Harris W.A., Tanaka H., Ohnuma S. (2004). Tsukushi functions as an organizer inducer by inhibition of BMP activity in cooperation with chordin. Dev. Cell.

[78] Wagener R., Ehlen H.W., Ko Y.P., Kobbe B., Mann H.H., Sengle G., Paulsson M. (2005). The matrilins--adaptor proteins in the extracellular matrix. FEBS Lett..

[79] Roman-Roman S., Garcia T., Jackson A., Theilhaber J., Rawadi G., Connolly T., Spinella-Jaegle S., Kawai S., Courtois B., Bushnell S. (2003). Identification of genes regulated during osteoblastic differentiation by genome-wide expression analysis of mouse calvaria primary osteoblasts in vitro. Bone.

[80] Filipp D., Alizadeh-Khiavi K., Richardson C., Palma A., Paredes N., Takeuchi O., Akira S., Julius M. (2001). Soluble CD14 enriched in colostrum and milk induces B cell growth and differentiation. Proc. Natl. Acad. Sci. USA.

[81] Manabe N., Kawaguchi H., Chikuda H., Miyaura C., Inada M., Nagai R., Nabeshima Y., Nakamura K., Sinclair A.M., Scheuermann R.H. (2001). Connection between B lymphocyte and osteoclast differentiation pathways. J. Immunol..

[82] Azuma K., Casey S.C., Ito M., Urano T., Horie K., Ouchi Y., Kirchner S., Blumberg B., Inoue S. (2010). Pregnane X receptor knockout mice display osteopenia with reduced bone formation and enhanced bone resorption. J. Endocrinol..

[83] Konno Y., Moore R., Kamiya N., Negishi M. (2010). Nuclear xenobiotic receptor PXR-null mouse exhibits hypophosphatemia and represses the Na/Pi-cotransporter SLC34A2. Pharm. Genom..

[84] Urano T., Shiraki M., Ouchi Y., Inoue S. (2007). Association of a single nucleotide polymorphism in the steroid and xenobiotic receptor (SXR) gene (IVS1-579A/G) with bone mineral density. Geriatr. Gerontol. Int..

[85] Neogi T., Booth S.L., Zhang Y.Q., Jacques P.F., Terkeltaub R., Aliabadi P., Felson D.T. (2006). Low vitamin K status is associated with osteoarthritis in the hand and knee. Arthritis Rheum..

[86] Oka H., Akune T., Muraki S., En-yo Y., Yoshida M., Saika A., Sasaki S., Nakamura K., Kawaguchi H., Yoshimura N. (2009). Association of low dietary vitamin K intake with radiographic knee osteoarthritis in the Japanese elderly population: Dietary survey in a population-based cohort of the ROAD study. J. Orthop. Sci..

[87] Misra D., Booth S.L., Tolstykh I., Felson D.T., Nevitt M.C., Lewis C.E., Torner J., Neogi T. (2013). Vitamin K deficiency is associated with incident knee osteoarthritis. Am. J. Med..

[88] Neogi T., Felson D.T., Sarno R., Booth S.L. (2008). Vitamin K in hand osteoarthritis: Results from a randomized clinical trial. Ann. Rheum. Dis..

[89] Azuma K., Casey S.C., Urano T., Horie-Inoue K., Ouchi Y., Blumberg B., Inoue S. (2015). Pregnane X receptor knockout mice display aging-dependent wearing of articular cartilage. PLoS ONE.

[90] Koike T., Izumikawa T., Tamura J., Kitagawa H. (2009). FAM20B is a kinase that phosphorylates xylose in the glycosaminoglycan-protein linkage region. Biochem. J..

[91] Oya K., Ishida K., Nishida T., Sato S., Kishino M., Hirose K., Ogawa Y., Ikebe K., Takeshige F., Yasuda H. (2017). Immunohistochemical analysis of dentin matrix protein 1 (Dmp1) phosphorylation by Fam20C in bone: Implications for the induction of biomineralization. Histochem. Cell Biol..

[92] Rosenberg I.H. (1997). Sarcopenia: Origins and clinical relevance. J. Nutr..

[93] Cruz-Jentoft A.J., Baeyens J.P., Bauer J.M., Boirie Y., Cederholm T., Landi F., Martin F.C., Michel J.P., Rolland Y., Schneider S.M. (2010). European Working Group on Sarcopenia in Older People. Sarcopenia: European consensus on definition and diagnosis: Report of the European Working Group on Sarcopenia in Older People. Age Ageing.

[94] Cruz-Jentoft A.J., Bahat G., Bauer J., Boirie Y., Bruyère O., Cederholm T., Cooper C., Landi F., Rolland Y., Sayer A.A. (2019). Writing Group for the European Working Group on Sarcopenia in Older People 2 (EWGSOP2), and the Extended Group for EWGSOP2. Sarcopenia: Revised European consensus on definition and diagnosis. Age Ageing.

[95] Shea M.K., Loeser R.F., Hsu F.C., Booth S.L., Nevitt M., Simonsick E.M., Strotmeyer E.S., Vermeer C., Kritchevsky S.B. (2016). Health ABC Study. Vitamin K Status and Lower Extremity Function in Older Adults: The Health Aging and Body Composition Study. J. Gerontol. A Biol. Sci. Med. Sci..

[96] Van Ballegooijen A.J., van Putten S.R., Visser M., Beulens J.W., Hoogendijk E.O. (2018). Vitamin K status and physical decline in older adults-The Longitudinal Aging Study Amsterdam. Maturitas.

[97] Shea M.K., Dawson-Hughes B., Gundberg C.M., Booth S.L. (2017). Reducing Undercarboxylated Osteocalcin With Vitamin K Supplementation Does Not Promote Lean Tissue Loss or Fat Gain Over 3 Years in Older Women and Men: A Randomized Controlled Trial. J. Bone Miner. Res..

[98] Fulton R.L., McMurdo M.E., Hill A., Abboud R.J., Arnold G.P., Struthers A.D., Khan F., Vermeer C., Knapen M.H., Drummen N.E. (2016). Effect of Vitamin K on Vascular Health and Physical Function in Older People with Vascular Disease—A Randomised Controlled Trial. J. Nutr. Health Aging.

